# Multifocal Οsteomyelitis Localization after Pyomyositis in Children: Importance of Timely Response

**DOI:** 10.7759/cureus.4463

**Published:** 2019-04-16

**Authors:** Stavros Angelis, Angelos Trellopoulos, Andreas K Kondylis, Hristos Mirtsios, Antonios Katsimantas, Evangelos P Solakis, Alexandros P Apostolopoulos, Zisis Kyriazis, John Ν Michelarakis

**Affiliations:** 1 Orthopedics, General Hospital Hellenic Red Cross Korgialenio - Benakio, Athens, GRC; 2 Orthopedics, General Children’s Hospital “Panagiotis & Aglaia Kyriakou”, Athens, GRC; 3 Anatomy and Surgical Anatomy, National and Kapodistrian University of Athens School of Medicine, Athens, GRC; 4 General Surgery, Evangelismos General Hospital, Athens, GRC; 5 Orthopedics, East Surrey Hospital, Surrey and Sussex Healthcare National Health Service Trust, Redhill, GBR

**Keywords:** pyomyositis, osteomyelitis, muscle abscess, infection, children

## Abstract

Pyomyositis is a rare bacterial infection that used to prevail in tropical areas for the past century. Nowadays though, more and more cases are reported in high-temperature climate areas. Diagnosis is often delayed due to the variance in clinical presentation, the challenging nature of physical examination of a child, and lack of specific laboratory investigating tools. When the diagnosis is delayed, the outcome may be unpredictable. Multifocal localization through hematogenous or direct spread that may affect the skeletal bone tissue is common. Timely diagnosis and response is a race against septic shock.

We present a case series of seven children diagnosed with pyomyositis due to Staphylococcus aureus. High or less clinical suspicion has obviously affected the final outcome since two patients who were not treated in time were subjected to a life-threatening hazard. Five patients who were diagnosed and treated within the first three days after initiation of their symptoms had a predictable and good outcome without complications.

## Introduction

Pyomyositis is an infection that affects large skeletal muscle groups and forms abscesses. Usually, the muscles of the lower limb and thigh region are affected. Quadriceps, iliopsoas and gluteal muscles are the most common location of primary myositis [1]. Staphylococcus aureus is the most common pathogen in children, followed by β-hemolytic streptococcus group A, Escherichia coli and Enterococcus [2,3]. Pathogenesis and risk factors of the disease have been proposed. These include intensive exercise and local trauma, malnutrition, viral and parasitic infections, bacteremia, immunodeficiency or chronic illness [4].

This infection used to prevail in tropical areas but nowadays, many cases are also reported in high-temperature climate areas. Diagnosis demands high clinical suspicion and delays may lead to high rates of morbidity and mortality. This may be a very difficult task, since there is variance in clinical presentation, difficulty and unclear results in the physical examination of a child, and lack of specific laboratory investigating tools. Imaging techniques should be recruited if there is suspicion. Magnetic resonance imaging (MRI) is the “gold standard” imaging technique, not only for the diagnosis but also for delineating the site of fluid concentration and the extent of bone tissue involvement.

Οsteomyelitis through direct spread from neighbouring muscle tissue as well as multifocal osteomyelitis through hematogenous spread is possible. We will present a series of primary pyomyositis infection in seven children, with or without secondary bone involvement. Our data suggest that timely attendance, high clinical suspicion and no delays in diagnosis are the main factors that could reduce morbidity and mortality rates.

## Materials and methods

Α retrospective study of all children (16 years old and under) treated for acute primary pelvic pyomyositis in our clinic, between January 2017 and December 2018, was performed.

We have included patients who presented with localized hip pain, limping, raised body temperature, swelling and oedema, muscle abscess or fluid in the radiology report. Only patients diagnosed to have primary pelvic pyomyositis in the medical record or radiology report were included. We excluded patients presented with primary osteomyelitis, primary septic arthritis, and secondary myositis after open trauma. Patients with chronic underlying illnesses were also excluded.

After determining the study group meeting the above criteria, patients' age, gender, symptoms and primary presenting complaint, preceding trauma or illness, laboratory results, pathogen grew in culture, duration of symptoms and treatment, need for surgical intervention, in-patient stay and follow-up were reviewed. Imaging from radiographs, ultrasonography (USG), bone scans, computerized tomography (CT) and MRIs were also reviewed.

Finally, we have categorized our patients into those who were treated timely and into those who were diagnosed or treated with a delay. We attempt to connect this to higher complication rates.

## Results

Pyomyositis was identified in 23 patients admitted to our hospital between January 2017 and December 2018. After excluding patients not meeting the sample's criteria, seven patients remained in the study group. Mean age was 8.9 years (range: three-year-old to 15-year-old) and male patients were more susceptible than female (6:1).

The main symptom concerning all patients was hip pain. High fever (>38.5°C) was also reported in all patients. Limping was very common (six out of seven) and so was the inability to weight bear (four out of seven). Swelling and oedema were obvious only in two patients, probably because of the deep site of affected muscles. One patient referred to our clinic from a provincial hospital, presented with cellulitis of the trochanteric area. Preceding hip trauma was reported by three patients and one had a history of otitis externa two weeks ago.

Laboratory screening was recorded at the peak of each inflammatory marker (Table 1). In all patients, the pathogen was identified. Blood cultures, abscess cultures, or both, revealed that all patients were infected by Staphylococcus aureus. In six patients, methicillin-sensitive Staphylococcus aureus (MSSA) was identified, and one patient was infected by methicillin-resistant Staphylococcus aureus (MRSA).

**Table 1:** Mean inflammatory markers, recorded when at their peak.

White blood cells (WBC)	18400/μl (range: 12800/μl – 28000/μl)
C-reactive protein (CRP)	232 mg/L (range: 183 mg/L – 356 mg/L)
Erythrocyte sedimentation rate (ESR)	96 mm/h (range: 70 mm/h – 135 mm/h)

We have noticed that according to the records, five of the patients were diagnosed and treated within the first three days after the initiation of their symptoms. One patient’s treatment began five days after the initiation of hip pain because of delay in seeking medical treatment by the parents. One patient began getting treated eight days after the beginning of his symptoms. He reported a hip trauma a few days before.

Group 1

**Figure 1:**
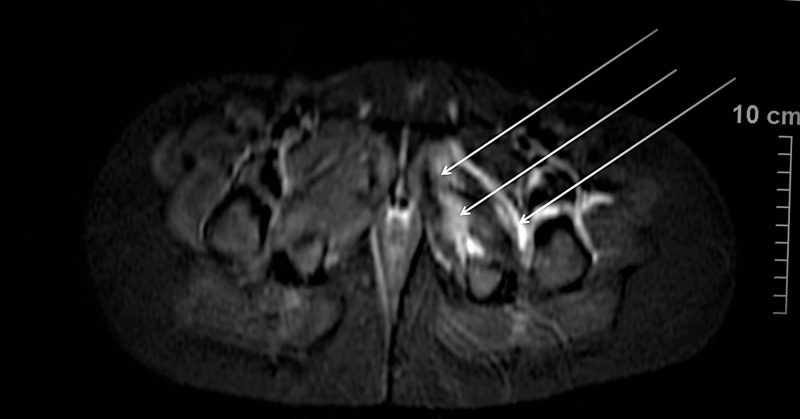
Magnetic resonance imaging (MRI) scan of a 10-year-old boy. Arrows point to the fluid concentration within or around muscles.

In the first group of the five patients who were treated timely, mean duration of symptoms until presentation, diagnosis and initiation of treatment was 2.2 days (range: one to three days). All patients performed radiographs and USGs on the day of their admission. USGs were usually repeated during their hospitalization as screening and proof of improvement. CT scan was performed in one patient two days after admission. Nuclear imaging scans were performed on three patients. All five patients performed MRI scans two or three days after admission (Figure 1). Intravenous broad-spectrum antibiotics were administered in all patients within a few hours of presentation. Once the pathogen was identified through blood cultures, the antibiotic treatment was adjusted according to the antibiogram. Only one patient had to undergo drainage of the abscess by the interventional radiologists of our hospital. Mean period of inpatient stay was 14 days (range: 10 to 20 days). Mean period of total antibiotic treatment, oral and intravenous, was 35 days (range: 28 to 42 days) and mean follow-up was 75 days (range: 60 to 90 days). All these patients recovered completely, as evidenced by clinical examination, C-reactive protein (CRP) screening or the repeat of USG and MRI scan.

Group 2

The two patients forming the second group were treated with a delay. The first patient (patient No. 1) was an eleven-year-old male and reported a history of otitis externa two weeks before initiation of his symptoms. Treatment began five days after the initiation of hip pain because of delay in seeking medical treatment by the parents. Until then, he also suffered from mild fever (up to 37.4°C) according to his parents. They were alerted when his fever grew higher (38.2°C). The second patient (patient No. 2), a 15-year-old boy, began getting treated eight days after the beginning of his symptoms, which were hip pain, limping, mild oedema of the hip and mild fever (up to 37.1°C). He reports a hip trauma five days before visiting a general practitioner in the island that he was passing the summer. His symptoms were attributed to the hip trauma and the parents were reassured. The boy was referred to our clinic from the local provincial hospital after he presented with fever and cellulitis over the left trochanteric and thoracic area.

**Figure 2:**
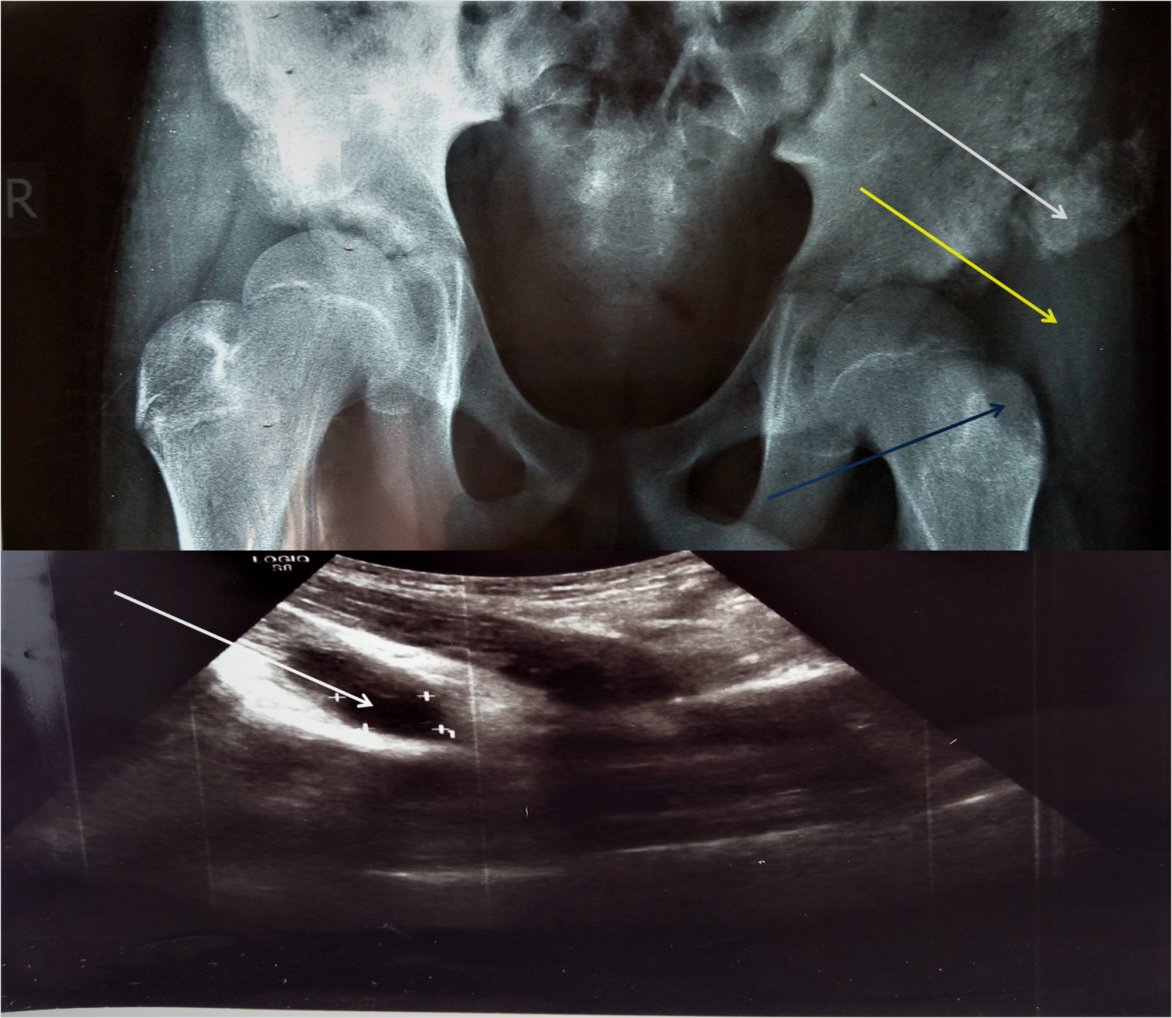
Radiograph and ultrasonography (USG) of patient No. 2. Calcification of a cystic formation (abscess) next to the left iliac wing (white arrows). USG report mentions a cystic formation (abscess) inside the left psoas muscle with dimensions of 3.94 cm x 2.01 cm. Swelling of soft tissue over the left trochanteric area (yellow arrow). Lytic lesion on the left trochanter (blue arrow).

**Figure 3:**
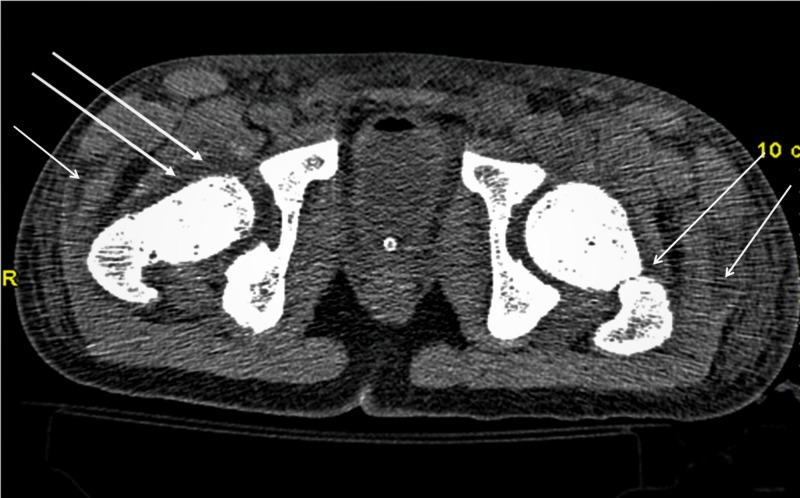
Computed tomography (CT) scan in patient No. 2 with pyomyositis located in both hip areas.

Both patients performed radiographs and USGs on the day of their admission. USGs and radiographs were repeated during their hospitalization as proof of improvement but also as part of a screening protocol for the proof of multifocal involvement of the disease (Figure 2). CT scan was performed in patient No. 2 showing multifocal pyomyositis on both hip areas (Figure 3). Bone scans were performed on both patients. Both patients were subjected to MRI scans twice during their inpatient stay.

Patient No. 1

**Figure 4:**
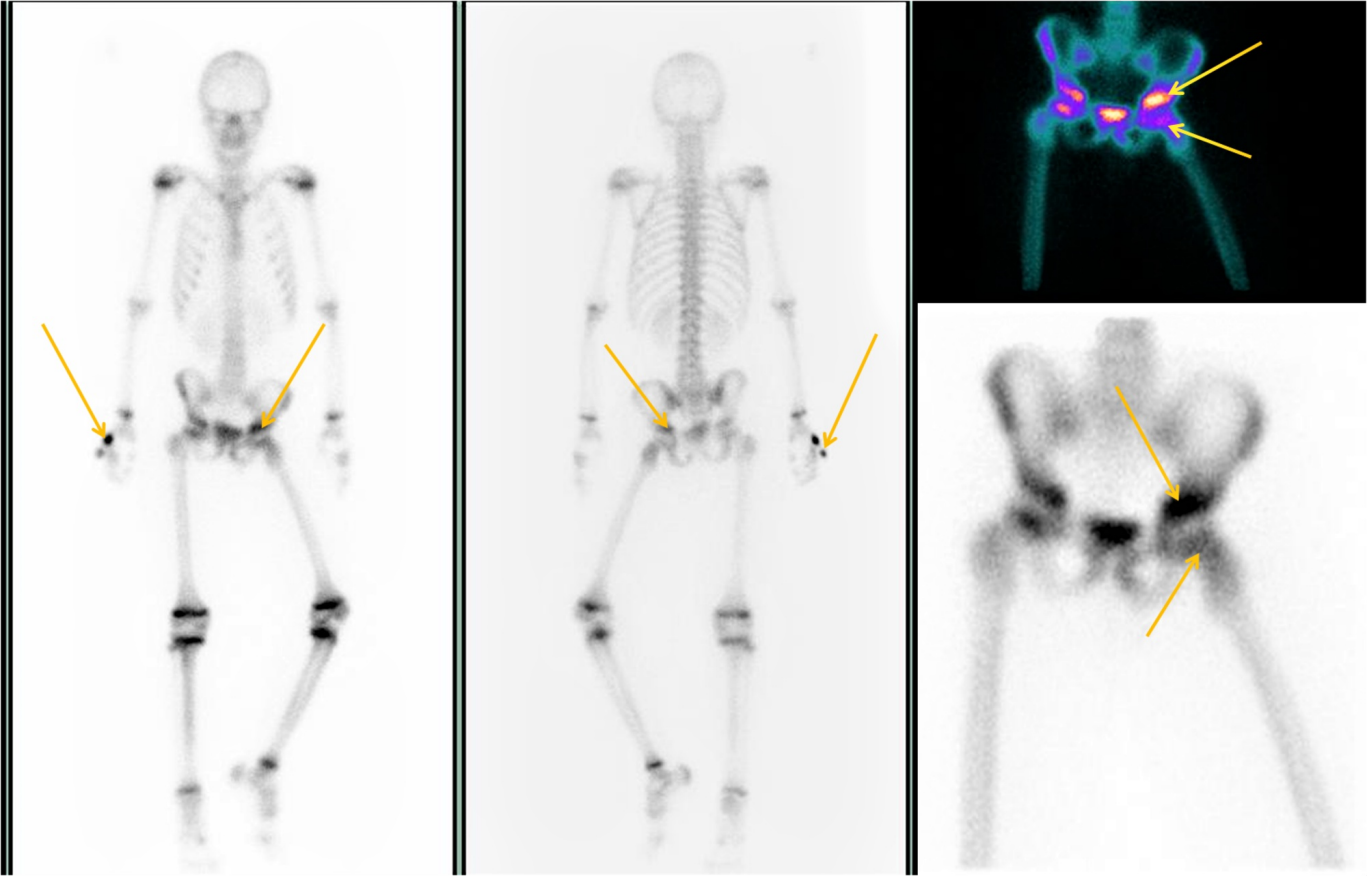
Bone scan of patient No. 1. Concentration at the left hip area and right small finger of the upper limb (arrows).

Patient No. 1 was admitted with the diagnosis of "pyomyositis of the left hip area". On the first day, drainage of abscess formation had to be performed. This was identified and guided by USG imaging. The delay in treatment had led to abscess formation. Intravenous broad-spectrum antibiotics were administered until the antibiotic treatment of abscess and blood cultures was adjusted according to the antibiogram. MRI scan on day two after admission revealed soft tissue but also bone and arthrosis involvement. Bone scan on day three, not only revealed soft tissue and bone involvement but also proved the involvement of the right small finger of the upper limb that was swollen and painful (Figure 4). The patient had to be subjected to a drainage procedure once more on day seven after admission, due to the persistence of fever and after a new USG imaging of the left hip area. The patient remained hospitalized for 27 days. Overall, he was under antibiotic treatment, oral and intravenous, for 42 days (six weeks) and was followed up for 90 days. Patient No. 1 recovered completely, as evidenced by clinical examination, CRP screening and a repeat of MRI scan.

Patient No. 2

**Figure 5:**
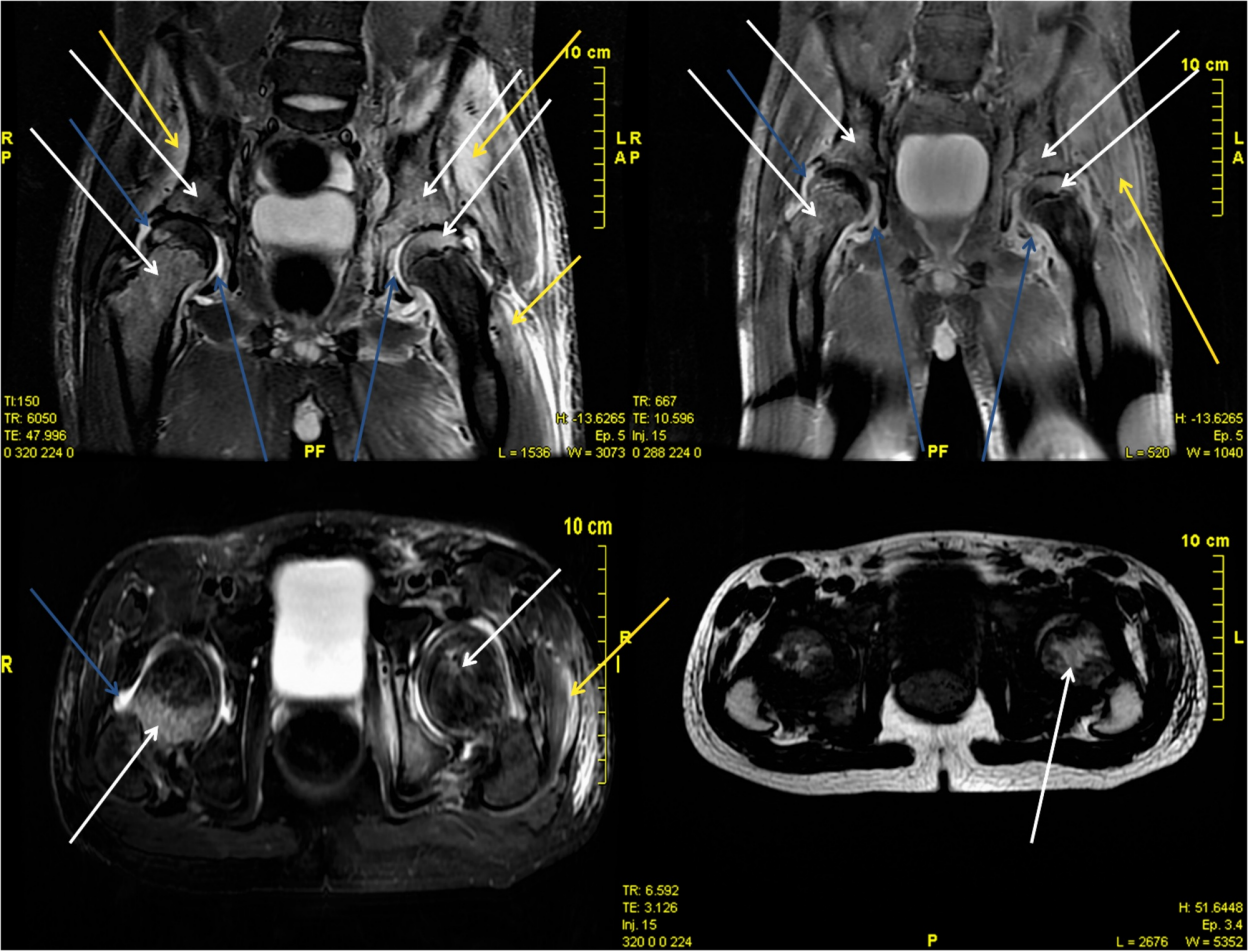
Magnetic resonance imaging (MRI) scan of patient No. 2. The white arrows point to bone involvement (osteomyelitis). The yellow arrows point to the soft tissue involvement (myositis). The blue arrows point to the arthrosis involvement (septic arthritis).

**Figure 6:**
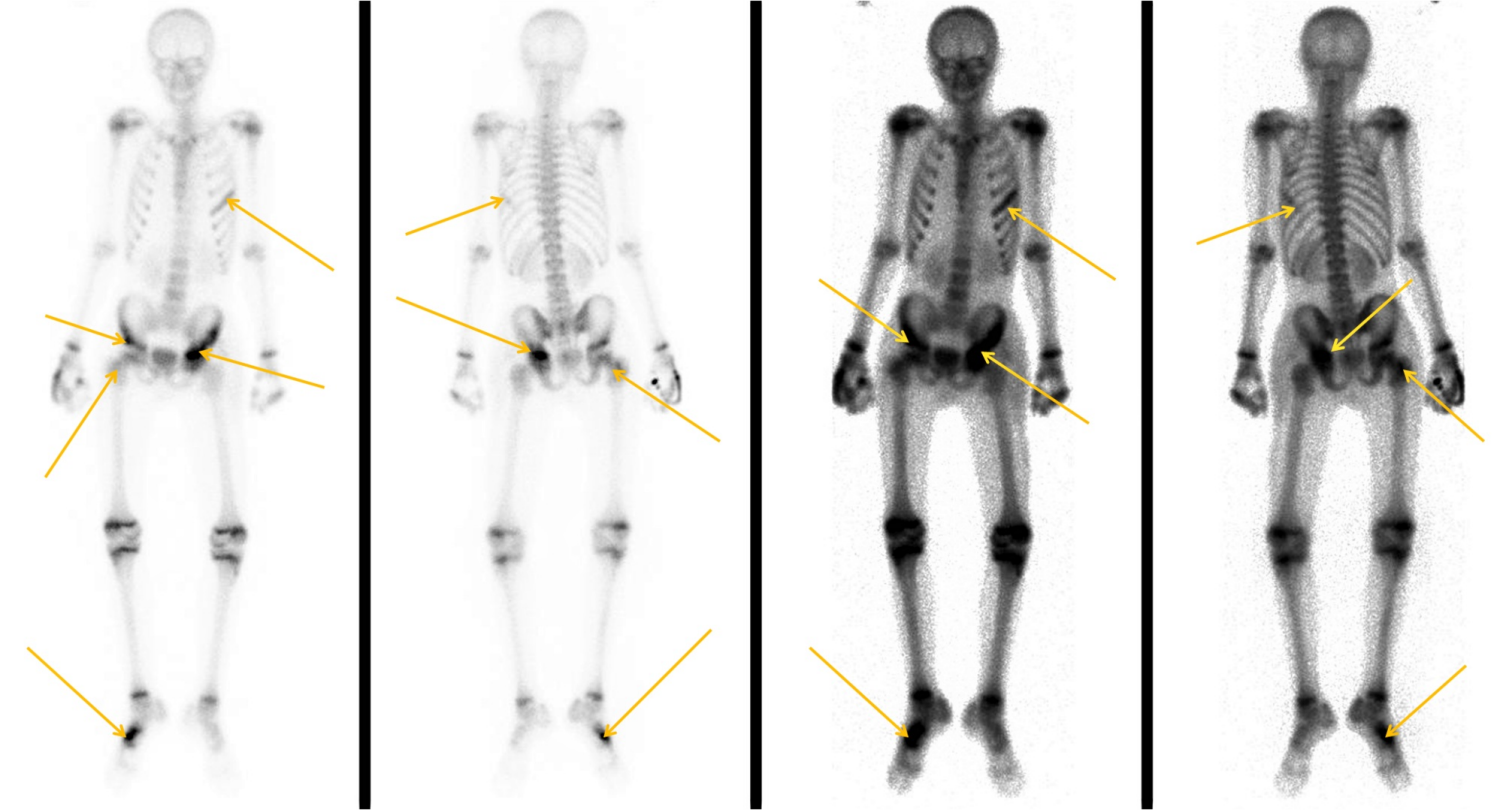
Bone scan of patient No. 2. The arrows point to the bone involvement.

**Figure 7:**
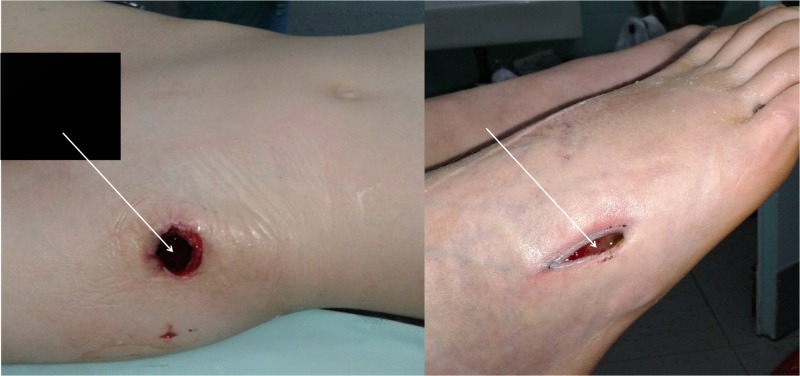
Wounds over the left hip and fifth right metatarsal for the drainage of the abscesses.

Patient No. 2 was admitted with the diagnosis "pyomyositis of the left hip area with cellulitis over the left trochanteric and left thoracic area". He also complained about pain and oedema over the fifth metatarsal bone of his right foot. Intravenous broad-spectrum antibiotics were administered until the antibiotic treatment was adjusted according to the antibiogram of the blood cultures received on day of admission. MRI scan performed on day two after admission, revealed soft tissue but also bone and arthrosis involvement of both hips (Figure 5). On day three after admission to our clinic the patient had to be admitted to the intensive care unit (ICU) because of sepsis. He remained there for six days. Delay in receiving treatment had led to a serious life-threatening condition. On day five after admission, a nuclear imaging scan was performed (during hospitalization in the ICU). This not only revealed soft tissue and bone involvement of both hip areas but also proved the involvement of the fifth right metatarsal bone and the fifth left rib (Figure 6). Abscess formation in the left hip area and the right foot had to be subjected to multiple drainage procedures, during and after hospitalization in the ICU (Figure 7). Negative-pressure wound therapy (vacuum pump using a foam dressing on the wound) was also applied to both areas. The patient was hospitalized for 48 days. Overall, patient No. 2 was under antibiotic treatment, oral and intravenous, for 63 days (nine weeks) and was followed up for six months. The patient used to report some mild pain and limping of the left hip for five months, but finally, no sign of morbidity is present. He has recovered completely, as evidenced by clinical examination, CRP screening and a repeat of MRI scan.

## Discussion

Pyomyositis is an infection that affects large skeletal muscle groups and forms abscesses. Usually, the muscles of the lower limb and thigh region are affected. There are three stages of presentation [3]. In the invasive stage, there may be diffuse pain with or without fever, caused by inflammation and oedema in the muscle. Mild leukocytosis may be present. An examination may reveal a swollen muscle, palpable abscesses or surrounding cellulitis. In the second stage (suppurative phase), there is localized intramuscular abscess formation with systemic symptoms. Pelvic pyomyositis can present with pain, inability to weight bear, fever and increased inflammatory markers. Aspiration of the affected area reveals purulent material. The majority of patients are diagnosed at this stage. In the third stage, extra muscular abscesses, osteomyelitis, septic arthritis, sepsis or toxic shock may be present.

Staphylococcus aureus is the most common pathogen in children, followed by β-hemolytic streptococcus group A, Escherichia coli and Enterococcus [2,3]. Pus culture positivity rates are often high. This usually gives the etiological agent. Blood culture positivity rates are often low [5,6]. Blood investigations may show anaemia, leukocytosis (shift to left) raised erythrocyte sedimentation rate (ESR) and CRP. Muscle enzymes are always normal [3,5]. Pathogenesis and risk factors of the disease have been proposed. These include intensive exercise and local trauma, malnutrition, viral and parasitic infections, bacteremia, immunodeficiency or chronic illness [3,4].

Diagnosis of this pathologic entity requires high clinical suspicion and may be difficult. It should be considered in any child presenting with fever (mild or high) and acute muscle pain, especially in the season of high temperature and high humidity and when a history of recent trauma exists [3]. At least the patient should be observed for the next few days by parents who should look for fever and worsening of symptoms. The role of the general practitioner or pediatrician in this is crucial.

Finally, diagnosis is proved through imaging procedures especially when there are fever and muscle pain, but no palpable mass. Simple radiographs may imply soft tissue swelling or even a widened fascial plane. This is usually not a reliable proof for diagnosis but it is useful in order to exclude any suspected bony pathology [3]. USG is used for diagnosis of muscle masses or cystic formations. It is also used to guide percutaneous aspiration for culture and drainage. It is noninvasive, easily obtainable and inexpensive [7]. Bone scans (nuclear imaging scans) may be useful in locating soft and bone tissue infections. It is more useful though in detecting multifocal sites of the disease. MRI is preferred to CT as it is able to delineate the extent of a muscle abscess. In many cases, the abscess is much larger than suspected by clinical examination and MRI has become the preferred procedure for establishing the diagnosis of pyomyositis [8-10].

After diagnosis, the next step is to cover the patient with broad-spectrum antibiotics. Antibiotic treatment is then adjusted according to the antibiogram. Abscess drainage depending on the size and symptoms may be necessary. In early stages, pyomyositis may be treated conservatively with antibiotics alone or antibiotics and percutaneous aspiration. However, surgical decompression may be required in 50% of cases [11]. Before discontinuing antibiotics, the patient should have documented normal levels of CRP and ESR [3]. In some cases follow-up with USG and MRI scans may be useful.

This pathologic entity is sometimes life-threatening if diagnosed late. Also, complications are common and directly related to timely treatment. These include venous thrombosis, septic arthritis, osteomyelitis, pneumonia, pericarditis, meningitis, sepsis and septic shock. Recurrence of fever after drainage when the patient is receiving appropriate antibiotic treatment suggests the presence of other abscesses needing further drainage, development of drug resistance or drug fever [3]. Timely diagnosis and treatment are usually equivalent to curing this potentially life-threatening situation.

## Conclusions

Pyomyositis is a rare bacterial infection that used to prevail in tropical areas for the past century. Nowadays though, increasing incidence in temperate regions is obvious. Staphylococcus aureus is the main pathogen. Timely diagnosis and treatment are the most important factors that affect the course of the disease. High level of suspicion is imperative for all clinicians dealing with children pathology.
